# Supplementary figures and images for: The genome formula of a multipartite virus is regulated both at the individual segment and the segment group levels
Source: PLoS Pathog. 2024 Jan 25;20(1):e1011973. doi: 10.1371/journal.ppat.1011973 (PMC10846721; doi:10.1371/journal.ppat.1011973)

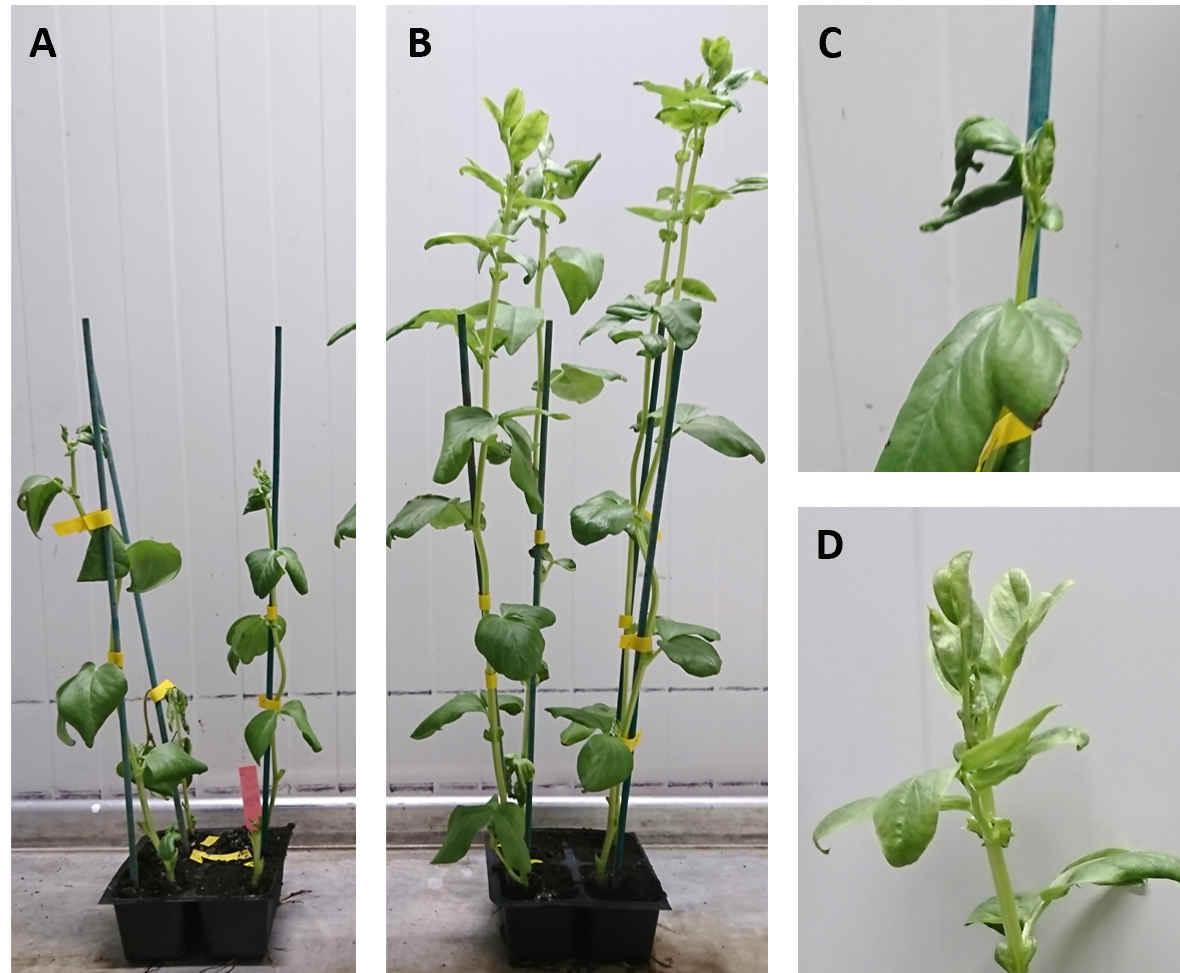

Supplement: S1 Fig — Symptoms of V. faba infected with FBNSVcomplete (A and C) or FBNSVU2- (B and D) four weeks after agro-inoculation. (TIF) [file ppat.1011973.s001.tif]

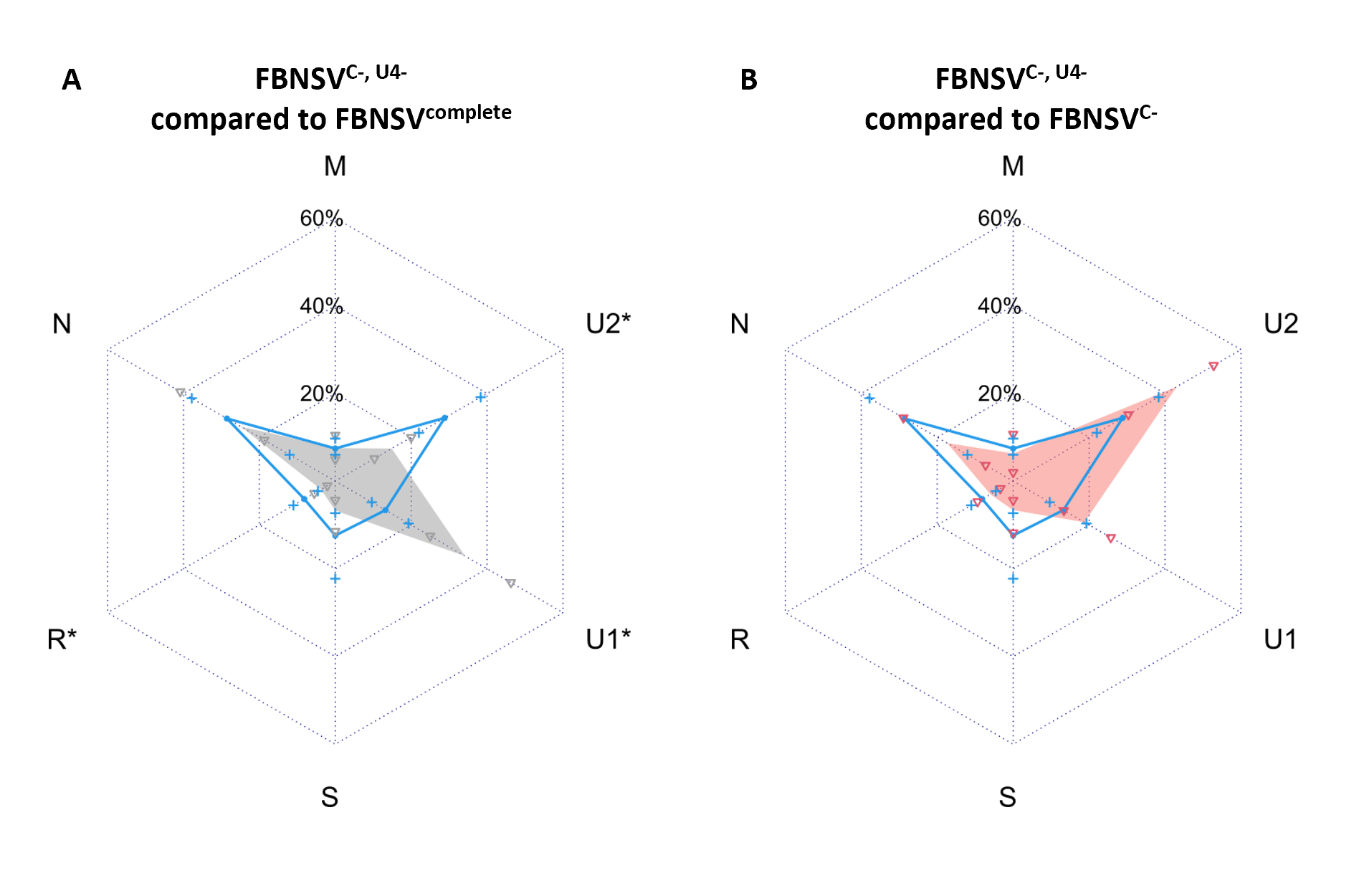

Supplement: S2 Fig — FBNSV genome formula in incomplete infections FBNSVC-, U4- (blue) compared to FBNSVcomplete (A; grey) or FBNSVC- (B; red). Genome segments accumulation in symptomatic V. faba plants was estimated by qPCR and the relative frequency of the segments was determined. To allow meaningful comparisons, the relative frequency of each segment was calculated without considering the accumulation of segments C and U4 in FBNSVcomplete and without considering U4 in FBNSVC-. Standard deviations are represented by grey triangles (complete infections) or red crosses (incomplete infections). Asterisks associated to segment names indicate when the differences in frequencies between complete and incomplete infections are statistically significant (Scheirer Ray Hare (p≤0.05) and post-hoc Dunn tests, Bonferroni correction). (TIF) [file ppat.1011973.s002.tif]

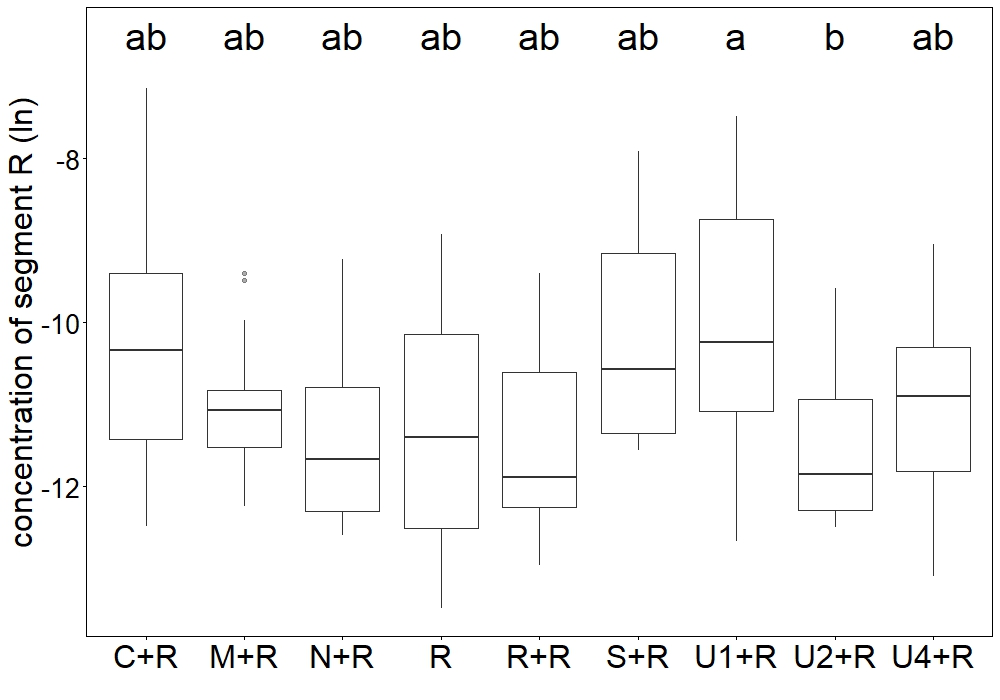

Supplement: S3 Fig — The accumulation of DNA-R when infiltrated with each of the seven other segments (C+R, M+R, N+R, S+R, U1+R, U2+R or U4+R) or alone with the same (R) or doubled (R+R) OD of infiltrated bacteria was determined by qPCR. For each box, the horizontal central bar represents the median and the edges of the rectangle the first and third quartiles. The vertical outer bars delineate the minimum and maximum values of the distribution, excluding outliers. The dots represent outliers. Letters above the boxes indicate segments with which DNA-R accumulation is statistically significant (Kruskal-Wallis tests and Bonferroni correction for multiple tests; S8 Table). (TIF) [file ppat.1011973.s003.tif]

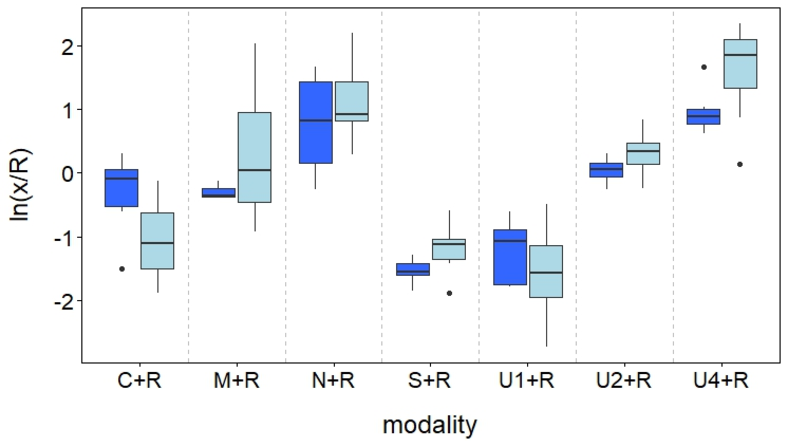

Supplement: S4 Fig — The relative accumulation ratio of each segment with respect to DNA-R obtained for the two experimental replicates are represented in dark and light blue respectively. For each box, the horizontal central bar represents the median and the edges of the rectangle the first and third quartiles. The vertical outer bars delineate the minimum and maximum values of the distribution, excluding outliers. The dots represent outliers. No statistically significant differences were found between the two experimental replicates (Scheirer Ray Hare test, p≤0.05; S9 Table). (TIF) [file ppat.1011973.s004.tif]
