# Supplementary material for: The genome formula of a multipartite virus is regulated both at the individual segment and the segment group levels
Source: PLoS Pathog. 2024 Jan 25;20(1):e1011973. doi: 10.1371/journal.ppat.1011973 (PMC10846721; doi:10.1371/journal.ppat.1011973)
Supplement: S1 Table — Comparisons of the accumulation of each segment relative to R to that of the others in systemically infected leaves were performed through Kruskal-Wallis tests using RStudio (package “agricolae”). The p-value indicating a statistically significant difference after Bonferroni correction (p≤0.05) is in red. (DOCX) [file ppat.1011973.s005.docx]

**S1 Table: Statistical analysis of the comparison of segment accumulation relative to R in systemically infected leaves.**

Comparisons of the accumulation of each segment relative to R to that of the others in systemically infected leaves were performed through Kruskal-Wallis tests using RStudio (package “agricolae”). The p-value indicating a statistically significant difference after Bonferroni correction (p≤0.05) is in red.

| **Source** | **DF** | **Chi-squared** | **p-value** |
| --- | --- | --- | --- |
| segment | 6 | 81.98917 | 1.332268e-15 |

| **Segment** | **Rank** | **Group** |
| --- | --- | --- |
| N | 103.73684 | a |
| U4 | 97.31579 | ab |
| U1 | 88.89474 | ab |
| U2 | 75.26316 | b |
| M | 46.42105 | c |
| C | 39.47368 | cd |
| S | 17.89474 | d |
