# Supplementary material for: The genome formula of a multipartite virus is regulated both at the individual segment and the segment group levels
Source: PLoS Pathog. 2024 Jan 25;20(1):e1011973. doi: 10.1371/journal.ppat.1011973 (PMC10846721; doi:10.1371/journal.ppat.1011973)
Supplement: S2 Table — Comparisons of the accumulation of each segment relative to R to that of the others in leaves infiltrated with pairs of segments were performed through Kruskal-Wallis tests using RStudio (package “agricolae”). The p-value indicating a statistically significant difference after Bonferroni correction (p≤0.05) is in red. (DOCX) [file ppat.1011973.s006.docx]

**S2 Table: Statistical analysis of the comparison of segment accumulation relative to R in leaves infiltrated with each segment in pair with R.**

Comparisons of the accumulation of each segment relative to R to that of the others in leaves infiltrated with pairs of segments were performed through Kruskal-Wallis tests using RStudio (package “agricolae”). The p-value indicating a statistically significant difference after Bonferroni correction (p≤0.05) is in red.

| **Source** | **DF** | **Chi-squared** | **p-value** |
| --- | --- | --- | --- |
| segment | 6 | 101.4495 | < 2.2e-16 |

| **Segment** | **Rank** | **Group** |
| --- | --- | --- |
| U4 | 109.86957 | a |
| N | 98.63158 | a |
| U2 | 75.61111 | b |
| M | 70.84615 | b |
| C | 41.45455 | c |
| S | 23.68750 | cd |
| U1 | 22.83333 | d |
|  |  |  |
