# Supplementary material for: The genome formula of a multipartite virus is regulated both at the individual segment and the segment group levels
Source: PLoS Pathog. 2024 Jan 25;20(1):e1011973. doi: 10.1371/journal.ppat.1011973 (PMC10846721; doi:10.1371/journal.ppat.1011973)
Supplement: S3 Table — For comparisons of the accumulation of each segment relative to R in each modality (systemic infections, leaf infiltration with pairs of segments or infiltrations with the eight segments), we first provide the output of a full model, ratio = modality * segment. This analysis was performed through Scheirer Ray Hare tests using RStudio (package “rcompanion”). After the full model tests we provide the output of per segment comparisons across pairs of modalities to identify segments whose relative frequency statistically significantly differed between modalities. The per segment differences were assessed through Dunn tests using RStudio (package “FSA”). The p-values indicating statistically significant differences after Bonferroni correction (p≤0.05) are in red. (DOCX) [file ppat.1011973.s007.docx]

**S3 Table: Statistical analysis of the comparison of segment accumulation according to the condition.**

For comparisons of the accumulation of each segment relative to R in each modality (systemic infections, leaf infiltration with pairs of segments or infiltrations with the eight segments), we first provide the output of a full model, ratio = modality * segment. This analysis was performed through Scheirer Ray Hare tests using RStudio (package “rcompanion”). After the full model tests we provide the output of per segment comparisons across pairs of modalities to identify segments whose relative frequency statistically significantly differed between modalities. The per segment differences were assessed through Dunn tests using RStudio (package “FSA”). The p-values indicating statistically significant differences after Bonferroni correction (p≤0.05) are in red.

Infiltrations with pairs of segments vs infiltrations with the eight segments

Full model:

| **Source** | **DF** | **Sum of Squares** | **H** | **p-value** |
| --- | --- | --- | --- | --- |
| segment | 6 | 309757 | 126.380 | 0.00000 |
| modality | 1 | 3472 | 1.416 | 0.23399 |
| segment*modality | 6 | 19316 | 7.881 | 0.24695 |
| residuals | 157 | 82134 |  |  |

Infiltrations with pairs of segments vs systemic infections

Full model:

| **Source** | **DF** | **Sum of Squares** | **H** | **p-value** |
| --- | --- | --- | --- | --- |
| segment | 6 | 896070 | 156.051 | 0.0000000 |
| modality | 1 | 21987 | 3.829 | 0.050370 |
| segment*modality | 6 | 195692 | 34.080 | 0.000006 |
| residuals | 248 | 393722 |  |  |

Per segment comparisons:

| **Segment** | **Z** | **p-value unadjusted** | **p-value adjusted** |
| --- | --- | --- | --- |
| C | -1.20264 | 0.2291156 | 1.000000e+00 |
| M | 1.055159 | 0.2913527 | 1.000000e+00 |
| N | -0.3649343 | 0.7151604 | 1.000000e+00 |
| S | -1.821232 | 0.0685716 | 4.800012e-01 |
| U1 | -5.196152 | 2.034555e-07 | 1.424189e-06 |
| U2 | -1.033153 | 0.3015323 | 1.000000e+00 |
| U4 | 3.247273 | 0.001165166 | 8.156162e-03 |

Infiltrations with the eight segments vs systemic infections

Full model:

| **Source** | **DF** | **Sum of Squares** | **H** | **p-value** |
| --- | --- | --- | --- | --- |
| segment | 6 | 250384 | 97.552 | 0.00000 |
| modality | 1 | 25772 | 10.041 | 0.0015308 |
| segment*modality | 6 | 50943 | 19.848 | 0.0029472 |
| residuals | 161 | 119501 |  |  |

Per segment comparisons:

| **Segment** | **Z** | **p-value unadjusted** | **p-value adjusted** |
| --- | --- | --- | --- |
| C | -1.399826 | 0.1615653 | 1.000000000 |
| M | -3.245052 | 0.001174291 | 0.008220037 |
| N | 2.354254 | 0.01855994 | 0.129919580 |
| S | -0.5726563 | 0.5668774 | 1.000000000 |
| U1 | -3.626823 | 0.0002869297 | 0.002008508 |
| U2 | -3.626823 | 0.0002869297 | 0.002008508 |
| U4 | 0.6999132 | 0.4839815 | 1.000000000 |
