# Supplementary material for: The genome formula of a multipartite virus is regulated both at the individual segment and the segment group levels
Source: PLoS Pathog. 2024 Jan 25;20(1):e1011973. doi: 10.1371/journal.ppat.1011973 (PMC10846721; doi:10.1371/journal.ppat.1011973)
Supplement: S5 Table — Infection rates and symptom severity were determined three (FBNSVcomplete, FBNSVN-, FBNSVU4-, FBNSVC- and FBNSVC-,U4-) or four (FBNSVU2-) weeks after inoculation. The presence of the segments was controlled by qPCR. (DOCX) [file ppat.1011973.s009.docx]

**S5 Table: Infection rates and phenotypes of FBNSV complete and incomplete infections in *V. faba.***

Infection rates and symptom severity were determined three (FBNSV^complete^, FBNSV^N-^, FBNSV^U4-^, FBNSV^C-^ and FBNSV^C-,U4-^) or four (FBNSV^U2-^) weeks after inoculation. The presence of the segments was controlled by qPCR.

| **Infection** | **Infection rates by agro-inoculation*** | **Symptoms** |
| --- | --- | --- |
| FBNSV^complete^ | 72/437 | Leaf curling, plant stunting |
| FBNSV^N-^ | 99/212 | Leaf curling, plant stunting |
| FBNSV^U4-^ | 84/178 | Leaf curling, plant stunting |
| FBNSV^C-^ | 38/428 | Leaf curling, plant stunting |
| FBNSV^U2-^ | 1/450** | Attenuated, slight reduction and deformation of the upper leaves, yellowing |
| FBNSV^C-,U4-^ | *** | Leaf curling, plant stunting |

* Plants for which segments other than those voluntarily omitted were absent were not counted as infected.

** Five batches of approximately 90 plants each were inoculated. Of these five batches, only one plant out of a batch of 89 plants had all seven inoculated FBNSV segments (without U2).

*** Plants infected with FBNSV^C-,U4-^  were inoculated with aphids, as explained in the Methods section, so there is no agro-inoculation infection rate.
