# Supplementary material for: The genome formula of a multipartite virus is regulated both at the individual segment and the segment group levels
Source: PLoS Pathog. 2024 Jan 25;20(1):e1011973. doi: 10.1371/journal.ppat.1011973 (PMC10846721; doi:10.1371/journal.ppat.1011973)
Supplement: S6 Table — For each type of incomplete infection, we first provide the output of a full model, frequency = segment * modality where modality corresponds to the incomplete vs complete infection treatments. After the full tests we provide the output of per segment comparisons across modalities to identify segments whose relative frequency statistically significantly differed between incomplete and complete infections. These analyses were performed through Scheirer Ray Hare tests and Dunn tests using RStudio (packages “rcompanion” and “FSA”). The p-values indicating statistically significant differences after Bonferroni correction (p≤0.05) are in red. (DOCX) [file ppat.1011973.s010.docx]

**S6 Table: Statistical analysis of the comparison of the segment relative frequency between complete and incomplete infections.**

For each type of incomplete infection, we first provide the output of a full model, frequency = segment * modality where modality corresponds to the incomplete vs complete infection treatments. After the full tests we provide the output of per segment comparisons across modalities to identify segments whose relative frequency statistically significantly differed between incomplete and complete infections. These analyses were performed through Scheirer Ray Hare tests and Dunn tests using RStudio (packages “rcompanion” and “FSA”). The p-values indicating statistically significant differences after Bonferroni correction (p≤0.05) are in red.

FBNSV^C-^

Full model:

| **Source** | **DF** | **Sum of Squares** | **H** | **p-value** |
| --- | --- | --- | --- | --- |
| segment | 6 | 1486291 | 187.403 | 0.00000 |
| modality | 1 | 1171 | 0.148 | 0.70083 |
| segment*modality | 6 | 236127 | 29.773 | 0.00004 |
| residuals | 294 | 711228 |  |  |

Per segment comparisons:

| **Segment** | **Z** | **p-value unadjusted** | **p-value adjusted** |
| --- | --- | --- | --- |
| M | 3.794806 | 0.0001477587 | 1.034311e-03 |
| N | 4.030509 | 5.565628e-05 | 3.895940e-04 |
| R | -2.781287 | 0.00541439 | 3.790073e-02 |
| S | -0.7071068 | 0.4795001 | 1.000000e+00 |
| U1 | 2.616295 | 0.00888897 | 6.222279e-02 |
| U2 | -4.855467 | 1.201036e-06 | 8.407252e-06 |
| U4 | -0.4242641 | 0.6713732 | 1.000000e+00 |

FBNSV^N-^

Full model:

| **Source** | **DF** | **Sum of Squares** | **H** | **p-value** |
| --- | --- | --- | --- | --- |
| segment | 6 | 2991663 | 241.572 | 0.00000 |
| modality | 1 | 30455 | 2.459 | 0.116842 |
| segment*modality | 6 | 169709 | 13.704 | 0.033127 |
| residuals | 371 | 1563693 |  |  |

Per segment comparisons:

| **Segment** | **Z** | **p-value unadjusted** | **p-value adjusted** |
| --- | --- | --- | --- |
| C | -0.4901895 | 0.6239998 | 1.000000e+00 |
| M | 4.682155 | 2.838753e-06 | 1.987127e-05 |
| R | 4.158159 | 3.208228e-05 | 2.245760e-04 |
| S | 1.656502 | 0.09762013 | 6.833409e-01 |
| U1 | -1.419859 | 0.1556487 | 1.000000e+00 |
| U2 | 2.721397 | 0.00650067 | 4.550469e-02 |
| U4 | -3.820097 | 0.0001333991 | 9.337937e-04 |

FBNSV^U2-^

Full model:

| **Source** | **DF** | **Sum of Squares** | **H** | **p-value** |
| --- | --- | --- | --- | --- |
| segment | 6 | 13303440 | 429.73 | 0.00000 |
| modality | 1 | 1316 | 0.04 | 0.83664 |
| segment*modality | 6 | 1983546 | 64.07 | 0.00000 |
| residuals | 595 | 3533857 |  |  |

Per segment comparisons:

| **Segment** | **Z** | **p-value unadjusted** | **p-value adjusted** |
| --- | --- | --- | --- |
| C | 5.448709 | 5.073684e-08 | 3.551579e-07 |
| M | -5.716327 | 1.088512e-08 | 7.619584e-08 |
| N | -4.570921 | 4.855862e-06 | 3.399103e-05 |
| R | 1.091883 | 0.2748847 | 1.000000e+00 |
| S | -2.547726 | 0.01084275 | 7.589925e-02 |
| U1 | -4.774311 | 1.803239e-06 | 1.262267e-05 |
| U4 | 5.694918 | 1.234316e-08 | 8.640212e-08 |

FBNSV^U4-^

Full model:

| **Source** | **DF** | **Sum of Squares** | **H** | **p-value** |
| --- | --- | --- | --- | --- |
| segment | 6 | 3554726 | 258.147 | 0.00000 |
| modality | 1 | 538 | 0.039 | 0.84328 |
| segment*modality | 6 | 92709 | 6.733 | 0.34629 |
| residuals | 392 | 1928944 |  |  |

FBNSV^C-, U4-^ compared to FBNSV^Complete^

Full model:

| **Source** | **DF** | **Sum of Squares** | **H** | **p-value** |
| --- | --- | --- | --- | --- |
| segment | 5 | 173385 | 91.860 | 0.00000 |
| modality | 1 | 1654 | 0.876 | 0.34925 |
| segment*modality | 5 | 37406 | 19.818 | 0.00135 |
| residuals | 138 | 68793 |  |  |

Per segment comparisons:

| **Segment** | **Z** | **p-value unadjusted** | **p-value adjusted** |
| --- | --- | --- | --- |
| M | -0.1664101 | 0.8678343 | 1.0000000000 |
| N | 0.2773501 | 0.7815113 | 1.0000000000 |
| R | -3.161791 | 0.00156802 | 0.0094081200 |
| S | -2.607091 | 0.009131511 | 0.0547890660 |
| U1 | 4.049311 | 5.136855e-05 | 0.0003082113 |
| U2 | -4.049311 | 5.136855e-05 | 0.0003082113 |

FBNSV^C-, U4-^ compared to FBNSV^C-^

Full model:

| **Source** | **DF** | **Sum of Squares** | **H** | **p-value** |
| --- | --- | --- | --- | --- |
| segment | 5 | 433652 | 124.434 | 0.00000 |
| modality | 1 | 6021 | 1.728 | 0.18869 |
| segment*modality | 5 | 24573 | 7.051 | 0.21688 |
| residuals | 192 | 243209 |  |  |
