# Supplementary material for: The genome formula of a multipartite virus is regulated both at the individual segment and the segment group levels
Source: PLoS Pathog. 2024 Jan 25;20(1):e1011973. doi: 10.1371/journal.ppat.1011973 (PMC10846721; doi:10.1371/journal.ppat.1011973)
Supplement: S7 Table — (DOCX) [file ppat.1011973.s011.docx]

**S7 Table: Sequences of the primers used to quantify segment accumulation by qPCR.**

| **Targeted segment** | **Primer forward (5’-3’)** | **Primer reverse (5’-3’)** |
| --- | --- | --- |
| C | GACCCATCAACTGAAGACCTGTTGTCTC | AGCAACTCTCTACATGCAAGGTCTTCCA |
| M | ACGCTGCGTATCAAGACGACGG | TCCCGGAAGATAGCATGCAAGCA |
| N | GTGGTTATCTGTATCATAATGATTATGGCT | CTTCATCTTTCTTCAAATACTGAGACT |
| R | GGAGAACTGAAGAAGAAGAAAGCTAT | AAGCAAAGCATCAACCTCG |
| S | AGAAAGATGACGTGTCTGGTAG | TCAGATAATTCACCAACTCTCCCG |
| U1 | AGCTATTGATTTGGAAGACAGAGT | CGACAGCCAAGAAGCGT |
| U2 | ATTAGAGTAGATGAATGCAAAACTTGTAT | TAAACCTTCTAGACTGCCATCGT |
| U4 | CCAGATTAAAGAAGTTACTGTCAGGTAATA | ATTCAGCATCCTCGAACGG |
