# Supplementary material for: The genome formula of a multipartite virus is regulated both at the individual segment and the segment group levels
Source: PLoS Pathog. 2024 Jan 25;20(1):e1011973. doi: 10.1371/journal.ppat.1011973 (PMC10846721; doi:10.1371/journal.ppat.1011973)
Supplement: S8 Table — Statistical analyses were performed through Kruskal-Wallis tests using RStudio (package “agricolae”). The p-value indicating a statistically significant difference after Bonferroni correction (p≤0.05) is in red. (DOCX) [file ppat.1011973.s012.docx]

**S8 Table: Statistical analysis of the comparison of DNA-R accumulation depending on the segment with which it is infiltrated.**

Statistical analyses were performed through Kruskal-Wallis tests using RStudio (package “agricolae”). The p-value indicating a statistically significant difference after Bonferroni correction (p≤0.05) is in red.

| **Source** | **DF** | **Chi-squared** | **p-value** |
| --- | --- | --- | --- |
| segment | 8 | 23.50942 | 0.002768223 |

| **Segment** | **Rank** | **Group** |
| --- | --- | --- |
| U1 | 97.77778 | a |
| S | 97.37500 | ab |
| C | 94.45455 | ab |
| M | 74.38462 | ab |
| U4 | 70.56522 | ab |
| R | 62.90000 | ab |
| N | 58.50000 | ab |
| 2xR | 58.30000 | ab |
| U2 | 53.16667 | b |
|  |  |  |
