# Supplementary material for: The genome formula of a multipartite virus is regulated both at the individual segment and the segment group levels
Source: PLoS Pathog. 2024 Jan 25;20(1):e1011973. doi: 10.1371/journal.ppat.1011973 (PMC10846721; doi:10.1371/journal.ppat.1011973)
Supplement: S9 Table — We provide the output of a full model, ratio = replicate * segment. Statistical analyses were performed through Scheirer Ray Hare tests using RStudio (package “rcompanion”). The p-values indicating statistically significant differences after Bonferroni correction (p≤0.05) are in red. (DOCX) [file ppat.1011973.s013.docx]

**S9 Table: Statistical analysis of the comparison of segment accumulation in infiltrations in pairs of segments across experimental replicates.**

We provide the output of a full model, ratio = replicate * segment. Statistical analyses were performed through Scheirer Ray Hare tests using RStudio (package “rcompanion”). The p-values indicating statistically significant differences after Bonferroni correction (p≤0.05) are in red.

Full model

| **Source** | **DF** | **Sum of Squares** | **H** | **p-value** |
| --- | --- | --- | --- | --- |
| segment | 6 | 137529 | 98.411 | 0.00000 |
| replicate | 1 | 103 | 0.074 | 0.78617 |
| segment*replicate | 6 | 6654 | 4.762 | 0.57473 |
| residuals | 115 | 30347 |  |  |
