# Supplementary material for: The genome formula of a multipartite virus is regulated both at the individual segment and the segment group levels
Source: PLoS Pathog. 2024 Jan 25;20(1):e1011973. doi: 10.1371/journal.ppat.1011973 (PMC10846721; doi:10.1371/journal.ppat.1011973)
Supplement: Attachment — Submitted filename: Bonnamy et al 2023-response to reviewers-R1.docx [file ppat.1011973.s014.docx]

**Point-by-point response to the reviewers’ comments**

(reviewers’ comments are in small italics while our responses are in blue normal text)

Reviewer #1:

*I am wondering if it may be worth mentioning that Agrobacterium tumefaciens is now known as Agrobacterium radiobacter. Line 364 – optical density should be all small letters.*

We thank Reviewer #1 for this update. We have accordingly amended the manuscript lines 369, 370, 373, 384 and 386. Line 378 – optical density has been written in small letters.

Reviewer #2:

Major comment:

*If I have understood the setup correctly, the authors infiltrate leaves with multiple transgenic agrobacterium strains, each of which introduces a genome segment. The authors also indicate at various points throughout the manuscript that the infiltration efficiency is variable, which is normal for this procedure. The authors therefore normalize the accumulation of the segment in question (call it X) with the level of accumulation of R. However, given that the two segments are being introduced by separate bacterial strains, this means the cells infiltrated by the two strains have different spatial distributions. Again, this point is acknowledged clearly by the authors, for example in the conclusion (e.g. lines 303-306, here referring to infiltrations with all genome segments). So that leaves us with uninfiltrated cells, fraction P(no R, no X), cells infiltrated with one of the two segments, fractions P(no R, X) or P(R, no X), and cells infiltrated with both segments, fraction P(R, X). The fractions P(no R, no X) and P(no R, X) will not contribute to accumulation, as R is required. Both the fractions P(R, no X) and P(R, X) will contribute to the measured accumulation, but the fraction P(R, no X) obviously only produces R and never X. The authors normalize the level of X by R, but that disregards the fact that only the fraction p(R, X) can contribute to the accumulation of X. Assuming random distributions, the fraction p(R, X) will be the product of subpopulations p(R)p(X). Therefore, changes in infiltration efficiency can have a much stronger effect on the fraction of p(R, X) than accounted for by a correction with p(R).*

*As a concrete example: imagine the accumulation of X is always equal to that of R in co-infiltrated cells. When we have a low level of infiltration efficiency (say < 50% of all susceptible cells for each bacterial strain), the fraction of p(R, X) is much smaller than the fraction p(R, no X), so by normalizing by R we grossly underestimate the actual accumulation of X in co-infiltrated cells (0.25 of the accumulation of R). When infiltration efficiency is very high (99%), after normalizing we get the correct value (~ 1).*

*In an ideal world, pairs of segments would be introduced by a single bacterial strain (introduces many complications) or an experimental estimate of the fraction p(R, X) would be obtained (also quite complex, e.g. requiring a separate treatment e.g. with fluorescent markers, leading to indirect comparisons). As neither of these options are very feasible, some remedies I can think of are: (i) show that infiltration efficiency is always relatively high and therefore its impact is limited, (ii) consider how much variation there is in R accumulation, and in particular whether this variation is segment dependent (where if it is segment dependent, it does complicate the interpretation of the results as there could be systematic biases which are amplified due to the reliance on the product p(R)p(X) in the observed accumulation), or (iii) provide some evidence the R protein is present in most of the infiltrated cells because of the viral gene expression product sharing (i.e., Sicard et al. 2019) (from reading the authors comments in the discussion, they do not believe this is the case). To be clear, to my mind this point does not invalidate the research, but it should be given careful consideration in the analysis and interpretation of the results, where possible.*

We thank Reviewer #2 for prompting us to further investigate the issue related with the uncertainty in completely controlling the efficiency of the viral segment replication through *Agrobacterium* infiltration within individual leaf cells. As suggested by Reviewer #2 we looked at whether the accumulation of the R segment varied depending on which the other segment it was infiltrated with. The answer is that overall it accumulates equally; the only statistically significant difference we found was between infiltrations with segment U1 (more accumulated) *vs*. with U2 (less accumulated). This analysis was added to the manuscript as S3 Fig (legend lines 607 to 614) and S8 Table (legend lines 659 to 662) and is commented lines 265 to 272.

Minor comments:

*1. I find the Author Summary (AS) a clear description of the research, as the abstract remains a little too abstract for me. Perhaps the authors can use the AS as a basis for the abstract?*

We modified the abstract to provide a clearer description of the research (lines 16 to 21).

*2. Lines 93-95: “The eight FBNSV segments are always found [...]”. That statement is undoubtedly correct, but what is the sample size it is based on? And consequently, how much weight can we lend to it?*

Precisions on source and sample size have been added lines 96 to 98.

*3. Related to the major comment and a discussion point: What about the effects of other segments on the accumulation of R? I would not be surprised if some viral protein-protein interactions do lead to e.g. a higher productivity of the M-Rep? Viral PPI are mentioned later in the discussion, but not in the section discussing differences in segment accumulation (lines 245-259).*

As stated above in our answer to the "major comment", DNA-R accumulates fairly equivalently regardless of the segment with which it is infiltrated. However, we agree that PPI could be involved in the differences in segment accumulation. We added this possibility to the discussion in lines 264 and 265.

*4. The GF reported in Figure 1 for systemic leaves correlates quite nicely* *with previous reports from this group. It may be worthwhile pointing this out, as I for one immediately want to cross-reference them.*

We have modified the text lines 132-134 to make this correlation clearer for the reader. We also added the information to the legend of Fig 1 (lines 160-161).

*5. Lines 282-283, point II: “the ABSENCE of interactions between the different segments beyond that with R” would account for the differences between the bilateral vs. communal comparisons? I would think its the opposite way around and that it is precisely these interactions that could account for the differences.*

We fully agree and tried to clarify the sentence line 296 .

*6. Just by looking at the GF data, there appears to be quite some heterogeneity in variances. However, ANOVA and Tukey posthoc tests are reported throughout the analysis. I see that these procedures are performed on transformed data, but I really wonder whether homoscedasticity assumptions are being met. Good to show this is the case and, if not, to adjust the procedure accordingly.*

We thank Reviewer #2 for highlighting the issue related with the homoscedasticity of our data. Indeed, even after transforming the data homoscedasticity, and in some cases normality of model residuals, were not respected. We thus adjusted the statistical procedures by using in all analyses non-parametric tests (Scheirer Ray Hare and Kruskal-Wallis tests) instead of the ANOVA parametric tests used in the previous analyses. This has resulted in some modifications to the statistical test results, which in no way alter the conclusions of this study (see below a list of the differences).

We added a paragraph in the statistical analysis section to explain the use of non-parametric tests (lines 428-432).

The supplemental tables and their legends showing the results of statistical analyses have been updated accordingly:

S1 to S4 Table (legends lines 622 to 645),

S6 Table (legend lines 650 to 657)

S9 Table (legend lines 663 to 667).

The name of the statistical method used has also been updated in:

- the material and method section: lines 4350-436, 441-444, 450-451 and 457-458
- the legend of the figures

Legend of Fig 1: lines 156-157 and 165-166

Legend of Fig 2: line 219

- the legend of the supplemental figures

Legend of S2 Fig: lines 605-606

Legend of S4 Fig: lines 620-621

With these non-parametric statistical tests, some statistical results differ from those of our previous analyses:

1) In the leaf infiltration experiment:

Comparison of segment accumulation relative to R in systemically infected leaves, leaves infiltrated with pairs of segments or with the eight segments: In systemic infections, segment C ratio is no more statistically different from that of R (S1 Table). In leaves infiltrated with the eight segments, segment U2 ratio is no more statistically different from that of U4 (S4 Table). The Fig 1A illustrating these results has been modified accordingly. These differences have no impact on our interpretation of the results.

Comparison of segment accumulation according to the condition (systemically infected leaves, leaves infiltrated with pairs of segments or with the eight segments): no statistically significant difference between the accumulation pattern of segments infiltrated in pairs vs. all eight segments was found with the non-parametric tests (S3 Table). This result further supports our conclusion that infiltrations do not reproduce the genome formula. Hence, the text has been modified lines 180-181 of the results and lines 313-314 of the discussion.

Comparison of segment accumulation in infiltrations in pairs of segments across experimental replicates: The non-parametric test did not detect any statistically significant difference between the two repetitions of the infiltration experiments (S9 Table, formerly called S8 Table in our first submission), which further supports our initial decision to pull the results of the two repetitions together. The S4 Fig (formerly called S3 Fig in our first submission) illustrating these results has been modified accordingly.

2) In the incomplete infection experiments:

Comparaison of FBNSV^N-^ and FBNSV^Complete^ genome formulas: in addition to those of the M, R and U2 segments already identified, the frequency of the U4 segment proved statistically different between the two types of infection with non-parametric tests. Fig 2A has been modified accordingly and the text has been adjusted line 203 of the Results section.

Comparaison of FBNSV^U4-^ and FBNSV^Complete^ genome formulas: No statistically significant differences between the two types of infection were found with the non-parametric tests, while the frequency of N was with the previous parametric tests. Fig 2B has been modified accordingly and the text has been adjusted lines 204 to 205 of the Results section.

Comparaison of FBNSV^C-^ and FBNSV^Complete^ genome formulas: frequency of segment U1 is no more statistically significantly impacted by the infection type. Fig 2C has been modified accordingly and the text has been adjusted line 226 of the Results section.

Comparaison of FBNSV^U2-^ and FBNSV^Complete^ genome formulas: frequency of segment S is no more statistically significantly impacted by the infection type. Fig 2D has been modified accordingly and the text has been adjusted line 228 of the Results section.

Comparaison of FBNSV^C-, U4-^ and FBNSV^Complete^ genome formulas: frequency of segment S is no more statistically significantly impacted by the infection type. S2A Fig has been modified accordingly.

Comparaison of FBNSV^C-, U4-^ and FBNSV^C-^ genome formulas: No statistically significant differences between the two types of infection were found with the non-parametric tests. S2B Fig has been modified accordingly and the text has been adjusted line 235 of the Results section.
